# Diversity of clinical isolates of *Aspergillus terreus* in antifungal susceptibilities, genotypes and virulence in *Galleria mellonella* model: Comparison between respiratory and ear isolates

**DOI:** 10.1371/journal.pone.0186086

**Published:** 2017-10-09

**Authors:** Eun Jeong Won, Min Ji Choi, Jong Hee Shin, Yeon-Jun Park, Seung A. Byun, Jee Seung Jung, Soo Hyun Kim, Myung Geun Shin, Soon-Pal Suh

**Affiliations:** 1 Department of Laboratory Medicine, Chonnam National University Medical School, Gwangju, Republic of Korea; 2 Department of Laboratory Medicine, The Catholic University of Korea College of Medicine, Seoul, Republic of Korea; Woosuk University, REPUBLIC OF KOREA

## Abstract

We analyzed the antifungal susceptibility profiles, genotypes, and virulence of clinical *Aspergillus terreus* isolates from six university hospitals in South Korea. Thirty one isolates of *A*. *terreus*, comprising 15 respiratory and 16 ear isolates were assessed. Microsatellite genotyping was performed, and genetic similarity was assessed by calculating the Jaccard index. Virulence was evaluated by *Galleria mellonella* survival assay. All 31 isolates were susceptible to itraconazole, posaconazole, and voriconazole, while 23 (74.2%) and 6 (19.4%) showed amphotericin B (AMB) minimum inhibitory concentrations (MICs) of ≤ 1 mg/L and > 4 mg/L, respectively. Notably, respiratory isolates showed significantly higher geometric mean MICs than ear isolates to AMB (2.41 *vs*. 0.48 mg/L), itraconazole (0.40 *vs*. 0.19 mg/L), posaconazole (0.16 *vs*. 0.08 mg/L), and voriconazole (0.76 *vs*. 0.31 mg/L) (all, *P* <0.05). Microsatellite genotyping separated the 31 isolates into 27 types, but the dendrogram demonstrated a closer genotypic relatedness among isolates from the same body site (ear or respiratory tract); in particular, the majority of ear isolates clustered together. Individual isolates varied markedly in their ability to kill infected *G*. *mellonella* after 72 h, but virulence did not show significant differences according to source (ear or respiratory tract), genotype, or antifungal susceptibility. The current study shows the marked diversity of clinical isolates of *A*. *terreus* in terms of antifungal susceptibilities, genotypes and virulence in the *G*. *mellonella* model, and ear isolates from Korean hospitals may have lower AMB or triazole MICs than respiratory isolates.

## Introduction

The importance of invasive fungal infections caused by *Aspergillus* species has increased among immunocompromised patients due to their considerably high rates of morbidity and mortality [1, 2]. Although *Aspergillus fumigatus* accounts for the majority of invasive aspergillosis, *Aspergillus terreus* has been recognized as an emerging pathogen of pneumonia, infective endocarditis, and disseminated infections with a high mortality rate [3–6]. Invasive pulmonary aspergillosis caused by *A*. *terreus* has been highlighted because it could rapidly progress in immunocompromised patients despite amphotericin B (AMB) therapy [3–6]. Invasive otomycosis by *A*. *terreus* can also develop in immunosuppressed patients, with lethal consequences if not treated properly [7, 8].

*In vivo* and *in vitro* data indicate that almost all *A*. *terreus* isolates are innately resistant to AMB [9–13]. However, unusually AMB-susceptible *A*. *terreus* isolates have become a focus of attention, and the relationship between the AMB susceptibility and virulence of *A*. *terreus* isolates was investigated using mouse or *Galleria mellonella* models [14–16]. Although these studies included fewer than five isolates from respiratory specimens, they showed that isolates with an AMB minimum inhibitory concentration (MIC) of 0.5 mg/L were more virulent than resistant isolates with an AMB MIC of > 4 mg/L [14–16].

A recent Korean surveillance study reported a high frequency of isolation of AMB susceptible isolates of *A*. *terreus* from ear cultures [17]. A different pattern of AMB susceptibility of *A*. *terreus* isolates could be related to a great genomic diversity in *A*. *terreus* [18]. Genetic diversity in *A*. *terreus* species has been demonstrated by genotyping methods, such as random amplified polymorphic DNA, repetitive-sequence-based polymerase chain reaction (PCR), inter-simple sequence repeat PCR, and multilocus sequencing typing [19]. However, these techniques have poor inter-laboratory reproducibility and do not allow for the exchange of results between laboratories [19]. Instead, microsatellite-based typing, also referred to as short tandem repeat (STR)-based typing, has been introduced for genotyping of *Aspergillus* species and could yield a precise numerical result. There are only three reports of genetic analysis of *A*. *terreus* based on STR typing [20–22]. Although *A*. *terreus* is a frequently encountered species based on Korean surveillance data [17], the molecular epidemiology of clinical *A*. *terreus* strains has not been investigated in Korea.

The aim of this study is to investigate the antifungal susceptibility profiles, genotypic relatedness, and virulence for clinical isolates of *A*. *terreus* obtained from Korean multicenter study. Microsatellite typing was performed using seven STR markers and virulence was evaluated by *G*. *mellonella* survival assay; moreover, the associations of the former two parameters with antifungal susceptibility and genotype were investigated. In addition, we compared the genotypic relatedness, antifungal susceptibility profiles and virulence of respiratory isolates of *A*. *terreus* recovered from Korean hospitals with those of ear isolates.

## Materials and methods

### Strains and antifungal susceptibility testing

A total of 31 clinical isolates of *A*. *terreus* from respiratory specimens (n = 15) and ear specimens (n = 16) were obtained from 6 South Korean university hospitals from January 2012 to August 2013. Species identification was performed by sequencing of the internal transcribed spacer (ITS) and β-tubulin regions [23, 24]. The MICs of AMB, itraconazole, posaconazole, voriconazole for each isolate were determined by the CLSI M38-A2 broth microdilution method [25]. The MIC results of isolates were analyzed using the following recently described epidemiological cutoff values (ECVs): AMB, 4 mg/L, itraconazole, 2 mg/L; posaconazole, 1 mg/L; and voriconazole, 2 mg/L [26]. Isolates for which the MICs were higher than the ECVs were defined as non-wild type (non-WT).

### Genotyping using microsatellite markers

In total of seven STR markers were used for microsatellite typing, with a modification of the method established by Rougeron et al. [20]. PCR was carried out using reaction conditions described previously with slight modification. Briefly, PCR involved an initial denaturation step of 95°C for 10 min followed by 30 cycles of 30 s denaturation, 30 s of annealing at 60°C and 1 min of extension at 72°C, with final elongation at 72°C for 10 min. The PCR products were diluted 10-fold with formamide and 1 μL of this diluted product was combined with 15 μL of formamide, with 0.3 μL of ROX 500 marker (Applied Biosystems Inc., Foster City, CA, USA). The amplicons were separated by size and detected on an ABI3130xL Genetic Analyzer platform equipped with a 16-capillary array (Applied Biosystems) as per the manufacturer’s recommendations. Repeat numbers in each marker were assigned by using *A*. *terreus* NIH 2624 as a reference. Genetic similarity was calculated according to the Jaccard-similarity coefficient J*xy* = *a*/(*a*+*b*+*c*), where J*xy* is the measurement of the genetic similarity between isolates *x* and *y*, *a* is the number of the same genotype in both isolates, *b* is the number of amplified products at each STR marker observed in *x* but not in *y*, and *c* is the number of amplified products at each STR marker observed in *y* but not in *x* [27]. The matrix of similarity was analyzed by the unweighted pairgroup method using the arithmetic average (UPGMA) and a dendrogram was constructed using PAUP software (ver. 4.0b10; David Swofford, Smithsonian Institution, Washington DC, USA).

### *In vivo* virulence study using *Galleria mellonella*

Virulence was evaluated in the insect model *G*. *mellonella* as described previously [14]. Briefly, groups of 20 larvae (~150 mg; S-worm, Cheonan, South Korea) were stored in wood shavings in the dark at 18°C prior to use. The following three control groups were included: larvae injected with 10 μL phosphate buffered saline (n = 20), larvae that received needle injury only (n = 20), and untouched larvae (n = 20). A Hamilton syringe (25 gauge, 50 μL) was used to inoculate larvae with *A*. *terreus* and for introduction of treatments or control solutions into the larvae. To determine the virulence of clinical *A*. *terreus* isolates, larvae were infected with 5 × 10^6^ conidia per larvae and survival was monitored up to 72 h post-infection at 37°C. Experiments were performed in duplicate and re-tested when the difference between the two experiments was > 15%. Data from all experiments were combined to calculate the mean values of percent survival values.

### Statistical analysis

Statistical anlyses were performed using GraphPad Prism 5 software (GraphPad Software Inc., La Jolla CA, USA). The Kruskal-Wallis test or Mann-Whitney test were used to determine differences in the survival at 72 h between groups. Survival rates were evaluated using Kaplan Meier survival curves and analyzed with the log rank (Mantel-Cox) method. Chi squared or Fisher’s exact tests were used to determine differences in the number or percentage of isolates between groups. Differences were considered significant at *P* < 0.05.

### Ethics statement

This study was approved by the Institutional Review Board of Chonnam National University Hospital (IRB CNUH-2015-112). A waiver of the requirement for informed consent was granted given the retrospective nature of the project. Patient information was anonymized and deidentified prior to analysis, and no information that could lead to patient identification was used.

## Results

### Antifungal susceptibility tests

For all 31 isolates, the MIC ranges of AMB, itraconazole, posaconazole, and voriconazole were 0.125–8 mg/L, 0.125–0.5 mg/L, 0.03–0.5 mg/L, and 0.125–2 mg/L, respectively (Table 1). Based on the CLSI ECVs, all isolates were susceptible to all antifungals tested except for AMB. The frequency of non-WT isolates for AMB (MIC > 4 mg/L) was 19.4% (6/31), while 74.2% (23/31) of *A*. *terreus* isolates showed low AMB MICs (0.125–1 mg/L). Overall, only six respiratory isolates were classified as non-WT to AMB and no ear isolate was non-WT for AMB. Respiratory isolates showed significantly higher geometric mean MICs for AMB than ear isolates (respiratory, 2.41 mg/L; ear, 0.48 mg/L), itraconazole (respiratory, 0.40 mg/L; ear, 0.19 mg/L), posaconazole (respiratory, 0.16 mg/L; ear, 0.08 mg/L), and voriconazole (respiratory, 0.76 mg/L; ear, 0.31 mg/L) (all, *P* <0.05).

**Table 1:** MICs of AMB and triazoles of 31 *A*. *terreus* isolates from respiratory and ear specimens.

Antifungal agents	Source	No. of isolates	Minimum inhibitory concentrations (MIC, mg/L) ^a^											Non-wild type isolates^b^, No. (%)
			0.015	0.03	0.06	0.125	0.25	0.5	1	2	4	8	Geometric mean	
Amphotericin B	Respiratory	15							8	1		6	2.41^c^	6 (40.0)
	Ear	16				2	3	7	3		1		0.48	0 (0.0)
	Total	31				2	3	7	11	1	1	6	1.05	6 (19.4)
Itraconazole	Respiratory	15					5	10					0.40^c^	0 (0.0)
	Ear	16				9	4	3					0.19	0 (0.0)
	Total	31				9	9	13					0.27	0 (0.0)
Posaconazole	Respiratory	15			4	3	6	2					0.16^c^	0 (0.0)
	Ear	16		1	11	2	2						0.08	0 (0.0)
	Total	31		1	15	5	8	2					0.11	0 (0.0)
Voriconazole	Respiratory	15						7	7	1			0.76^c^	0 (0.0)
	Ear	16				2	9	3	2				0.31	0 (0.0)
	Total	31				2	9	10	9	1			0.48	0 (0.0)

^a^Antifungal MICs were determined by the method described in the CLSI M38-A2 document [25].

^b^CLSI ECVs were used to classify the strains as wild type (WT) or non-wild type (non-WT) in terms of susceptibility [26].

^c^Geometric mean MICs for the four antifungal agents differed significantly between the respiratory and ear isolates (all, *P* <0.005).

### Genotyping using microsatellite markers

**Fig 1:**
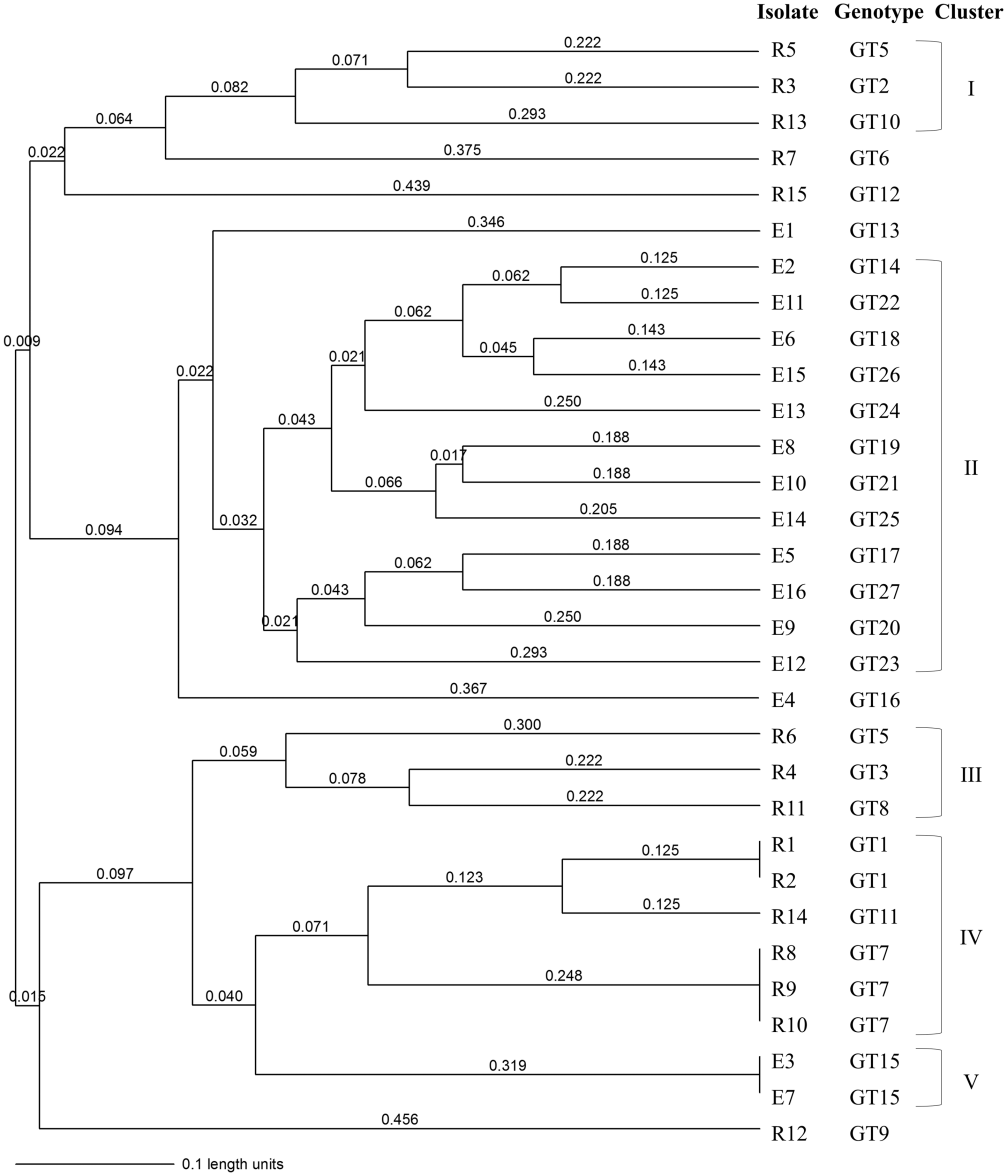
Genetic relationships of 31 *A*. *terreus* isolates according to source. The dendrogram is based on a categorical analysis of seven microsatellite markers in combination with unweighted pairgroup method using the arithmetic average (UPGMA) clustering. The number on the tree indicates the branch length, showing the difference along a branch. All 31 isolates (R1 to R15 and E1 to E16) comprised 27 distinct genotypes (GT 1 to GT 27) by 7 microsatellite markers. However, when a cluster is defined as the isolation of two or more strains with a branch length distance of < 0.63, ear isolates comprise clusters II and V, and the respiratory isolates comprise clusters I, III, and IV, suggesting a closer genetic relatedness among isolates from the same body site (ear or respiratory tract). Five isolates (R7, R15, E1, E4, and R12) were unique to a single isolate, which did not cluster with other isolate as a branch length distance of < 0.63. See Table 2 for detailed information on each isolate.

A total of 31 *A*. *terreus* isolates were subjected to microsatellite typing using nine STR markers for genotype determination. However, two STR markers (3A and 3C) were excluded because loci 3A showed limited allelic variation (n = 3), and 27 of 31 (87.1%) isolates failed to amplify for loci 3C. Therefore, seven of nine STR markers were ultimately used for genotype determination in this study. Overall, the 31 isolates comprised 27 distinct genotypes: 24 genotypes (88.8%) were unique to a single isolate, and 3 genotypes (genotypes 1, 7, and 15) were shared by 7 isolates (11.2%) (Table 2). Two strains of genotype 1, both of which were AMB non-WT (MIC > 4 mg/L), were isolated from respiratory specimens of two patients who were hospitalized in the same hospital (hospital A) at a similar time. Of the three respiratory strains of genotype 7, two were isolated from the same hospital (hospital C), but 6 months apart. Two strains of genotype 15 were isolated from ear specimens from two different hospitals (hospital C and F). Overall, the 15 respiratory isolates yielded 12 genotypes and the 16 ear isolates yielded 15 genotypes, respectively. The genetic similarity of the 31 isolates was determined by calculating the Jaccard index. The values ranged from 0.0 to 1.0, with a mean ± SD of 0.18 ± 0.20. The average genetic similarity value was 0.33 ± 0.19 for the 16 ear isolates, and 0.22 ± 0.25 for the 15 respiratory isolates, which were significantly higher than that of the 31 isolates (0.18 ± 0.20) (*P* < 0.05) (Table 3). The average genetic similarity value of the ear isolates did not differ significantly from that of the respiratory isolates. The *A*. *terreus* isolates exhibited high genetic diversity at 7 loci by microsatellite analysis (Fig 1). However, the dendrogram for the 31 isolates demonstrated a closer genotypic relatedness among isolates from the same body site (ear or respiratory tract); in particular, the majority of the ear isolates clustered together.

**Table 2:** Antifungal susceptibilities, microsatellite genotypes and virulence of 31 *A*. *terreus* isolates from six hospitals in South Korea.

Isolate No.	Isolation	Hos	Sources	Antifungal agent MIC (mg/L)				Microsatellite typing^a^		*Galleria mellonella* survival rate (%)^b^
	Year-Month-Day			AMB	ITRA	POSA	VOR	Profiles of 7 microsatellite markers	Combined genotype	
**R1**	2013-03-07	A	Respiratory	8	0.5	0.06	0.5	122-136-122-126-112-144-150	Genotype 1	10.0
**R2**	2013-03-21	A	Respiratory	8	0.5	0.06	0.5	122-136-122-126-112-144-150	Genotype 1	23.1
**R3**	2013-03-26	B	Respiratory	8	0.25	0.125	1	125-136-138-118-110-116-148	Genotype 2	12.5
**R4**	2013-04-04	B	Respiratory	8	0.25	0.125	0.5	122-132-130-134-132-144-150	Genotype 3	30.0
**R5**	2013-06-25	C	Respiratory	8	0.5	0.25	1	125-128-138-118-110-168-148	Genotype 4	27.5
**R6**	2013-06-25	C	Respiratory	8	0.5	0.25	1	122-132-130-126-130-144-148	Genotype 5	47.5
**R7**	2012-10-25	C	Respiratory	2	0.5	0.25	2	137-128-138-118-114-144-148	Genotype 6	52.5
**R8**	2012-02-10	D	Respiratory	1	0.5	0.25	1	122-132-122-126-126-144-150	Genotype 7	25.0
**R9**	2012-10-25	C	Respiratory	1	0.25	0.25	0.5	122-132-122-126-126-144-150	Genotype 7	45.0
**R10**	2013-08-16	C	Respiratory	1	0.25	0.125	0.5	122-132-122-126-126-144-150	Genotype 7	10.0
**R11**	2012-02-16	D	Respiratory	1	0.5	0.25	1	122-132-130-134-132-150-148	Genotype 8	28.3
**R12**	2012-03-07	D	Respiratory	1	0.5	0.5	1	119-132-122-130-130-142-152	Genotype 9	35.0
**R13**	2012-06-25	C	Respiratory	1	0.25	0.06	0.5	125-136-142-126-110-116-148	Genotype 10	50.0
**R14**	2012-07-20	C	Respiratory	1	0.5	0.06	0.5	122-136-130-126-112-144-150	Genotype 11	25.0
**R15**	2013-04-09	D	Respiratory	1	0.5	0.5	1	128-136-142-118-118-132-154	Genotype 12	27.5
**E1**	2013-03-21	E	Ear	4	0.125	0.06	0.5	125-116-130-146-108-176-148	Genotype 13	5.0
**E2**	2013-03-19	E	Ear	1	0.125	0.06	0.25	128-116-130-146-110-124-150	Genotype 14	7.5
**E3**	2013-05-22	F	Ear	1	0.5	0.25	1	122-136-130-126-136-144-176	Genotype 15	10.0
**E4**	2013-06-25	C	Ear	1	0.125	0.06	0.25	131-116-130-146-108-128-182	Genotype 16	72.5
**E5**	2012-06-05	C	Ear	0.5	0.125	0.03	0.25	128-116-130-158-110-172-148	Genotype 17	65.0
**E6**	2012-07-17	C	Ear	0.5	0.25	0.06	0.5	128-116-130-146-108-180-150	Genotype 18	37.5
**E7**	2012-07-26	C	Ear	0.5	0.5	0.06	1	122-136-130-126-136-144-176	Genotype 15	40.0
**E8**	2012-10-04	C	Ear	0.5	0.125	0.06	0.25	128-116-130-146-108-172-148	Genotype 19	10.0
**E9**	2012-10-15	C	Ear	0.5	0.25	0.125	0.25	128-116-130-158-110-134-152	Genotype 20	35.0
**E10**	2012-10-19	C	Ear	0.5	0.125	0.06	0.125	128-116-130-146-110-150-148	Genotype 21	20.0
**E11**	2013-08-12	C	Ear	0.5	0.125	0.06	0.25	128-116-130-146-110-184-150	Genotype 22	25.0
**E12**	2012-09-02	F	Ear	0.25	0.5	0.125	0.5	128-116-130-162-108-162-148	Genotype 23	70.0
**E13**	2012-09-11	C	Ear	0.25	0.25	0.06	0.25	128-116-130-146-110-176-146	Genotype 24	30.0
**E14**	2012-10-02	C	Ear	0.25	0.25	0.25	0.25	128-116-142-146-110-172-148	Genotype 25	70.0
**E15**	2012-09-12	C	Ear	0.125	0.125	0.06	0.25	128-116-130-146-108-150-150	Genotype 26	52.5
**E16**	2012-12-31	E	Ear	0.125	0.125	0.06	0.125	128-116-130-158-108-182-148	Genotype 27	10.0

Abbreviations: AMB, amphotericin B; Hos, hospital; ITR, itraconazole; MIC, minimum inhibitory concentrations; POS, posaconazole; VOR, voriconazole

^a^By microsatellite strain typing, each strain was characterized using the sizes of the amplified products of seven microsatellite markers (3B-4A-4B-4C-2A-2B-2C).

^b^To determine the virulence potential of *A*. *terreus* clinical isolates, *G*. *mellonella* larvae were infected with 5 × 10^6^ *A*. *terreus* conidia and percent survival was evaluated at 72 h post-infection.

**Table 3:** Genetic relatedness by microsatellite analysis and virulence in the *G*. *mellonella* model of 15 respiratory and 16 ear isolates of *A*. *terreus* from six hospitals.

	Respiratory isolates (n = 15)	Ear isolates (n = 16)	Total isolates (n = 31)
**Average genetic similarity in microsatellite typing, Mean ± SD (ranges)** ^a^	0.22 ± 0.25 (0.0–1.0)^b^	0.33 ± 0.19 (0.0–1.0)^c^	0.18 ± 0.20 (0.0–1.0)
**Survival rate (%) of infected *G*. *mellonella* at 72h, Mean ± SD (ranges)**	29.9 ± 13.84 (22.26–37.59)	35.0 ± 24.46 (21.97–48.03)	32.6 ± 19.88 (25.25–39.84)
**Isolates showing the lower survival rate of infected *G*. *mellonella* than mean value, No. (%)**^d^	10 (66.7) ^b^	8 (50.0)	18 (58.1)

^a^ Genetic similarity was calculated according to the Jaccard-similarity coefficient: Jxy = a/(a+b+c), where Jxy is a measurement of the genetic similarity between isolates x and y, a is the number of the same genotype in both isolates, b is the number of amplified products of each STR marker observed in x but not in y, and c is the number of amplified products of each STR marker observed in y but not in x.

^b^ *P* < 0.05, respiratory vs. ear isolates.

^c^ *P* < 0.05, ear vs. total isolates.

^d^The number of *A*. *terreus* isolates with a *G*. *mellonella* survival rate lower than the mean for the total of 31 isolates (32.6%).

### Virulence study using *Galleria mellonella*

**Fig 2:**
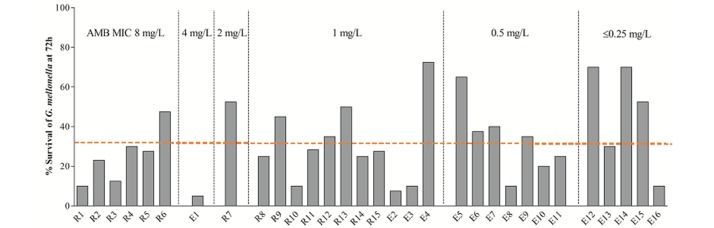
Survival rate (%) of *Galleria mellonella* larvae 72 h after inoculation with respiratory (R1 to R15) or ear (E1 to E16) *A*. *terreus* isolates. The 31 clinical *A*. *terreus* isolates exhibited marked diversity of virulence in *G*. *mellonella* model. Dotted horizontal bar indicates the mean value of the 31 isolates (32.6%). See Table 2 for detailed information on each isolate.

The mean survival rates of *G*. *mellonella* infected with each of the 31 *A*. *terreus* isolates was 32.6%, with a range of 5% to 72.5% (Table 2, Fig 2). There was no significant difference in the mean survival rate of *G*. *mellonella* infected with *A*. *terreus* according to the increment of AMB MIC [38.8% (10.0–70.0%) for an MIC of 0.125–0.5 mg/L; 30.3% (5.0–72.5%) for an MIC of 1–4 mg/L; and 25.1% (10.0–47.5%) for an MIC of 8 mg/L; *P =* 0.3902]. There was no significant difference in the survival rate of *G*. *mellonella* infected with non-WT and WT isolates (25.1% vs. 34.3%, non-WT vs. WT isolates, *P =* 0.0967). Also, the mean survival rate of *G*. *mellonella* did not differ according to the source of the infecting larvae (35.0% vs. 29.9%, ear vs. respiratory isolates, *P* = 0.6227) (Table 3 and S1 Fig). Virulent isolates, which resulted in a lower survival rate of *G*. *mellonella* than the overall mean survival rate (32.6%) were more frequent among respiratory versus ear isolates (50.0% vs. 66.7%, ear vs. respiratory isolates, *P* <0.05).

## Discussion

This study characterized the antifungal susceptibilities, genetic relatedness, and virulence in a *G*. *mellonella* model of clinical *A*. *terreus* isolates obtained during laboratory-based surveillance of six Korean hospitals. Although *A*. *terreus* is known to be intrinsically resistant to AMB [9–13], our findings suggest that AMB susceptible clinical isolates of *A*. *terreus* are not uncommon, especially in ear specimens. Notably, we report for the first time that isolates from the same body site (ear or respiratory tract) have greater genetic similarity, and respiratory isolates are significantly less susceptible to AMB and triazoles than ear isolates. However, there was no difference in virulence between respiratory and ear isolates in a *G*. *mellonella* model.

Many investigations show that *A*. *terreus* has intrinsic resistance to AMB, with elevated MICs [9–13], but others have reported that *A*. *terreus* isolates have a wide spectrum of AMB MIC ranging from 0.125 mg/L to 16 mg/L [18, 28, 29]. Our results also showed the broad distribution of AMB MICs, ranging from 0.125 to 8 mg/L. According to the most recent report, 12–13% of *A*. *terreus* isolates worldwide have low AMB MICs (≤ 1 mg/L) [10, 11, 21]. In the present study, 74.2% (23/31) of the isolates had an AMB MIC of ≤ 1 mg/L, suggesting that AMB susceptibility is not uncommon among clinical *A*. *terreus* isolates in Korea. The high proportion of AMB susceptible *A*. *terreus* isolates in the present study may be due to the high proportion of ear isolates in this Korean collection. Ear isolates showed significantly lower geometric mean AMB MICs (0.48 mg/L) than respiratory isolates (2.41 mg/L). The respiratory isolates exhibited AMB MICs of 0.5 to 8 mg/L, in line with other reports [30, 31].

Of two previous studies using microsatellite typing, one reported a high genetic diversity in a large collection of geographically diverse *A*. *terreus* isolates from clinical and environmental sources in India [21], while the other suggested the existence of geographically predominant genotypes of *A*. *terreus* species complex in Austria [22]. In the present study, using the microsatellite typing method, the 31 isolates comprised 27 distinct genotypes, indicating that, with the exception of two Genotype 1 isolates, the *A*. *terreus* isolates in our collection are highly diverse and unlikely to originate from a common source. Why *A*. *terreus* isolates from ear cultures are more susceptible to AMB and triazoles than those from respiratory cultures is unclear, but the explanation may involve the genetic relatedness of the isolates. Notably, the average genetic similarity value of ear isolates was significantly higher than that of the total 31 isolates. The average genetic similarity value of respiratory isolates was also significantly higher than that of the total 31 isolates. Taken together, these findings suggest that Korean *A*. *terreus* isolates exhibit considerable genetic diversity without a predominant genotype, but ear isolates from South Korean hospitals comprise closely related genotypes with lower AMB or triazole MICs than respiratory isolates.

After Rougeron et al. [20] introduced microsatellite-based typing using nine markers for *A*. *terreus*, a multicenter study used this method to conduct genotyping on clinical and environmental isolates from India, North America and Europe [21]. In that study using nine markers, however, 17.2% (21/122) of isolates revealed no amplification at >2 loci (of all nine loci) after repeated attempts; therefore, 21 isolates were excluded from their study [21]. When we performed typing using all nine microsatellite markers, no isolates had amplification at >2 loci. Instead, all but four isolates showed no amplification at one locus (3C) after repeated attempts, and all but three showed the same genotype (93 in size) at locus 3A. These results may, in part, be influenced by the composition of isolates within a collection with geographic differences. Therefore, in the present study, of the nine microsatellite markers used, two (3A and 3C) were excluded. Overall, a combination of seven markers revealed that the 31 isolates comprised 27 distinct genotypes, and the dendrogram for the 31 isolates demonstrated a closer genotypic relatedness among isolates from the same body site (ear or respiratory tract); in particular, the majority of ear isolates clustered together, suggesting successful microsatellite testing using seven markers for all isolates of *A*. *terreus* from South Korean hospitals.

The virulence of AMB susceptible strains of *A*. *terreus* has been a focus of interest [14–16]. In an *in vivo* murine model of disseminated aspergillosis, AMB-susceptible strains (MIC, 0.5 mg/L) were more virulent than AMB-resistant strains (MIC, 4 mg/L) [14]. Similarly, in the *G*. *mellonella* model, two AMB-resistant strains showed lower virulence than three AMB-susceptible strains [16]. These results are in agreement with previous findings in a murine model [15], suggesting that *G*. *mellonella* facilitates *in vivo* screening of *A*. *terreus*. In the present study, the virulence of 31 clinical isolates of *A*. *terreus* in the *G*. *mellonella* model varied markedly, which confirms a previous report that AMB susceptibility in *A*. *terreus* is not necessarily associated with loss of *in vivo* virulence [14]. However, there was no statistically significant difference in the average 72-h survival rate of *G*. *mellonella* infected with AMB-susceptible and -resistant isolates (MIC ≤ 0.5 mg/L and > 4 mg/L, respectively) (Fig 2). Rather, a trend toward a lower survival rate of infected *G*. *mellonella* according to the increment of AMB MIC (38.8% for a MIC of 0.125–0.5 mg/L; 30.3% for a MIC of 1–4 mg/L; and 25.1% for a MIC of 8 mg/L, respectively) was observed. The reasons for these discrepant results are unclear, but the results might have been influenced by the composition of the isolates. One possible explanation is that the previous three studies tested a few selected isolates, but we assessed 31 clinical *A*. *terreus* isolates with a wide range of AMB MICs. Another possible explanation is the AMB-susceptible strains (AMB MIC, 0.5 mg/L) tested in the previous three studies were from respiratory cultures, while all AMB-susceptible strains in the present study were from ear cultures because all respiratory isolates had an AMB MIC ≥ 1 mg/L.

Although *A*. *terreus* is found frequently in the environment [32], infections caused by *A*. *terreus* are less common than those caused by *A*. *fumigatus* [5]. However, *A*. *terreus* infections are associated with a lower rate of a response to AMB therapy and are more frequently fatal than *A*. *fumigatus* infections [10]. Here we report for the first time that the antifungal susceptibilities, genotypes, and virulence of clinical *A*. *terreus* isolates vary markedly, and that respiratory isolates comprise diverse genotypes with higher AMB or triazole MICs than ear isolates. Because the main portal of entry and site of infection for *Aspergillus* in human hosts is the respiratory tract, respiratory isolates of *A*. *terreus* may be more likely to cause severe infections than ear isolates. Although we did not find a difference in virulence between respiratory and ear isolates, a significantly higher proportion of respiratory isolates than ear isolates was able to kill infected *G*. *mellonella*. These characteristics of *A*. *terreus* isolates were also supported by clinical information (S1 Table). All patients with ear isolates presented with otorrhea; but had a relatively good prognosis. Of 15 patients with respiratory isolates, 10 had evidence of lung lesions or bronchopulmonary infections, such as pneumonic infiltration, bronchiolitis, or tuberculous empyema, and 6 died mainly due to therapeutic failure from treatment with azoles or caspofungin. There would be several factors that contribute to aspergillosis, including both fungus and host related factors such as strain virulence and host pulmonary structure/immune status, respectively [33]. Therefore, given the marked diversity of *A*. *terreus* strains and their opportunistic behavior, we postulate that strain-specific factors contribute to colonization of different body sites, or to aspergillosis, depending on the patient population.

## Supporting information

S1 Fig Kaplan Meier survival curves of 31 *Aspergillus terreus* isolates included in this study. Overall survival did not differ significantly according to specimen type (A) or amphotericin B resistance (B).
(TIF)

S1 Table Clinical information of patients with 31 *Aspergillus terreus* isolates included in this study. (PDF)
