# Supplementary figures and images for: Diversity of clinical isolates of Aspergillus terreus in antifungal susceptibilities, genotypes and virulence in Galleria mellonella model: Comparison between respiratory and ear isolates
Source: PLoS One. 2017 Oct 9;12(10):e0186086. doi: 10.1371/journal.pone.0186086 (PMC5633196; doi:10.1371/journal.pone.0186086)

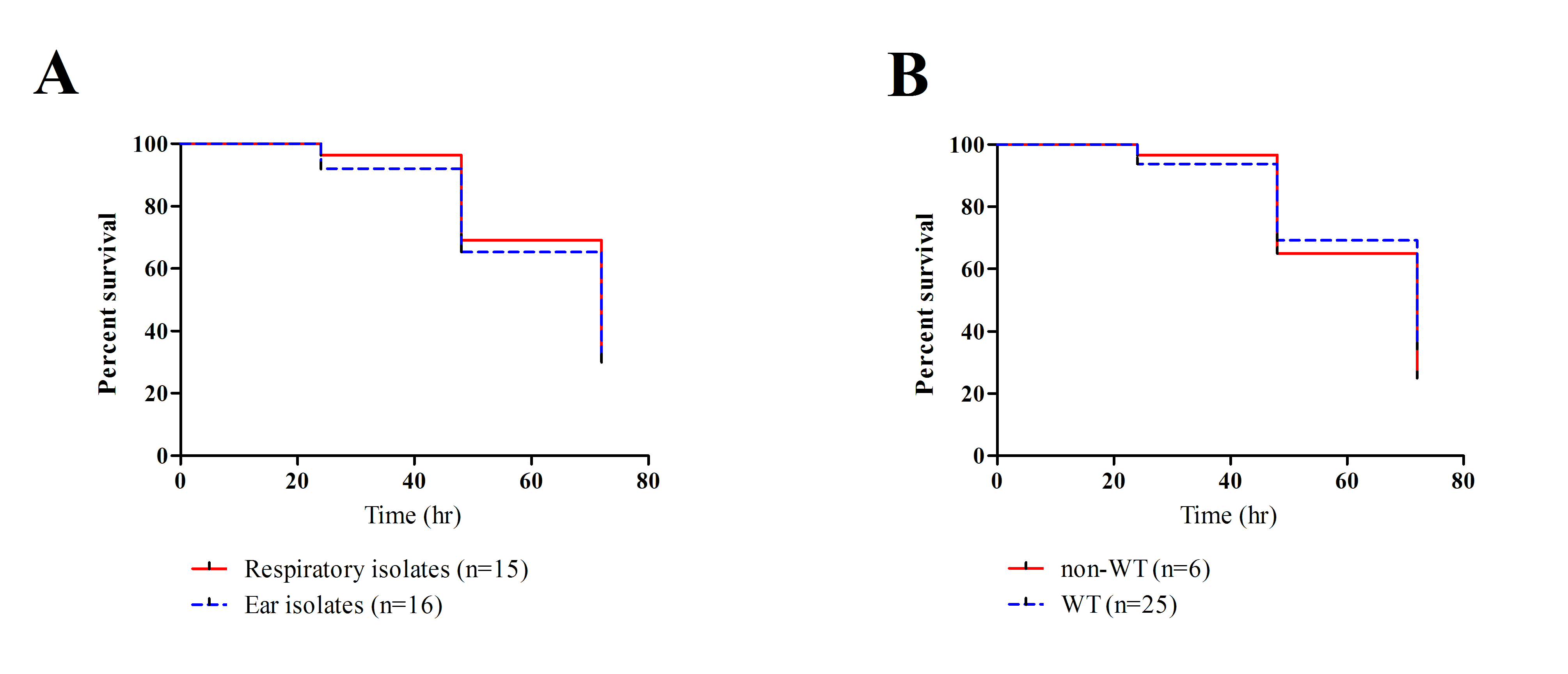

Supplement: S1 Fig — Overall survival did not differ significantly according to specimen type (A) or amphotericin B resistance (B). (TIF) [file pone.0186086.s001.tif]
