# Supplementary material for: Diversity of clinical isolates of Aspergillus terreus in antifungal susceptibilities, genotypes and virulence in Galleria mellonella model: Comparison between respiratory and ear isolates
Source: PLoS One. 2017 Oct 9;12(10):e0186086. doi: 10.1371/journal.pone.0186086 (PMC5633196; doi:10.1371/journal.pone.0186086)
Supplement: S1 Table — (PDF) [file pone.0186086.s002.pdf]

S1 Table. Clinical information of patients with 31 *Aspergillus terreus* isolates included in this study

Isolate No.	Age(yrs) /Sex	Underlying disease	Prior antifungal agents exposure	Radiological findings[a]	Antifungal therapy after diagnosis	Patient's outcome
R1	63/M	Diabetes mellitus, Lung mass	No	No	No	Survive
R2	84/M	Gastric cancer, End stage kidney disease on dialysis	No	No	No	Survive
R3	63/M	Kidney transplantation	Itraconazole	Pneumonic infiltrations	Caspofungin, voriconazole, itraconazole, fluconazole	Death
R4	73/M	Lung cancer with multiple LN metastasis	No	Tuberculosis empyema	No	Survive
R5	69/M	Liver cirrhosis	No	Pneumonic infiltrations	Voriconazole	Death
R6	68/M	Liver cirrhosis	No	NA	Voriconazole	Death
R7	79/M	Lumbar spine fracture	No	NA	No	Survive
R8	24/F	Hematologic malignancy, Pneumonia	Fluconazole	Pneumonic infiltrations	Caspofungin	Death
R9	68/F	Hypertension	No	NA	No	Survive
R10	67/M	Lung cancer	No	NA	No	Survive
R11	79/M	Hematologic malignancy, Pneumonia	No	Pneumonic infiltrations	No	Survive
R12	69/M	Chronic obstructive pulmonary disease	No	Pneumonic infiltrations	No	Survive
R13	79/M	Chronic obstructive pulmonary disease	No	NA	No	Death
R14	72/F	Idiopathic pulmonary fibrosis	No	NA	Voriconazole	Death
R15	57/F	Hematologic malignancy,	No	Bronchiolitis	No	Survive

		sibling PBSCT				
E1	55/F	Otorrhea	NA	No	No	Survive
E2	60/M	Otorrhea	NA	No	No	Survive
E3	66/M	Otitis media externa	No	NA	No	Survive
E4	34/M	Pneumonia, otorrhea	No	NA	Voriconazole	Survive
E5	53/M	Chronic otitis media	No	NA	No	Survive
E6	53/M	Nasopharyngeal cancer	No	NA	No	Survive
E7	33/F	Chronic otitis media	No	NA	No	Survive
E8	54/F	Otorrhea	No	NA	No	Survive
E9	57/M	Otorrhea	No	NA	No	Survive
E10	33/M	Otorrhea	No	NA	No	Survive
E11	69/F	Chronic otitis media	No	NA	No	Survive
E12	32/F	Attic cholesteatoma	No	No	Itraconazole	Survive
E13	55/F	Chronic otitis media	No	NA	No	Survive
E14	66/F	Otorrhea	No	NA	No	Survive
E15	38/M	Otorrhea	No	NA	No	Survive
E16	51/M	Otorrhea	NA	No	No	Survive

[a] ‘NA (not available)’ indicates that a radiological examination was not performed.
